# Twelve Tips for Creating and Supporting a Meaningful Asynchronous Learning as Parts of Virtual Transition of a Curriculum

**DOI:** 10.15694/mep.2021.000111.1

**Published:** 2021-05-05

**Authors:** Surbhi Maheshwari, Parag Jain, Betty Lee Ligon, Satid Thammasitboon

**Affiliations:** 1 Texas Children's Hospital; 2 Baylor College of Medicine

**Keywords:** community of inquiry, blended learning, curriculum development, learning technology.

## Abstract

This article was migrated. The article was marked as recommended.

With increasing use of online learning, the focus is rapidly shifting from in-person learning to virtual asynchronous learning (ASL). The flexibility offered by ASL coupled with advancements in technologies have enabled increased engagement of learners. However, ASL has inherent challenges associated with it, especially for educators. Successful curricula designed for virtual learning should deliver high-quality education, leverage existing technologies, and incorporate evidence-informed educational practices. Contrariwise, if the discourse becomes diluted due to lack of clear goals and objectives, it can lead to identity challenges in educators and dissatisfaction among learners. In this contemporary review, we offer practical tips for educators to guide development and/or implementation of curricula in asynchronous platforms using a Community of Inquiry framework. The guide also elaborates on practical use of various technologies to conform with one’s teaching identity as identified by Pratt’s teaching perspectives.

## Introduction

Successful curricula designed for virtual learning should deliver high-quality education, leverage existing technologies, and incorporate evidence-informed educational practices. With the advent of modern technology and software, most teaching institutions have introduced virtual learning experiences for their students. The need for virtual learning was amplified during the COVID-19 pandemic, shifting the focus from in-person classroom teaching to a virtual one ( Gordon *et al.,* 2020). The latter inherently adds complexity to the education process and often compromises learning previously achieved in an in-person classroom. Hence, medical educators and program developers need to develop expertise in the use of virtual asynchronous learning (ASL) that offers flexibility for both educators and learners to deliver and receive the curricular content, respectively.

A good example is Massive Online Open Course (MOOC), an open-access, online curriculum aimed at an unlimited number of participants. It provides opportunities for networked learning across several platforms and services, thereby developing shared practices, knowledge, and understanding ( Yuan, Powell and Olivier, 2014). Contrariwise, if not done properly, ASL does not provide the social-cultural phenomenon associated with learning. Thus, it is important for educators to be mindful of its limitations and incorporate educational principles and best practices when designing an effective ASL experience. We have integrated advice in the contemporary literature with the Community of Inquiry (CoI) framework developed by Garrison and colleagues ( Garrison, Anderson and Archer, 1999), along with insights from Pratt’s teaching styles ( Pratt and Collins, 2020), to offer 12 Tips for creating an effective ASL experience that includes social engagement, with the aim of helping other medical educators develop successful ASL curricula.

## Conceptual Framework

**Figure 1.**
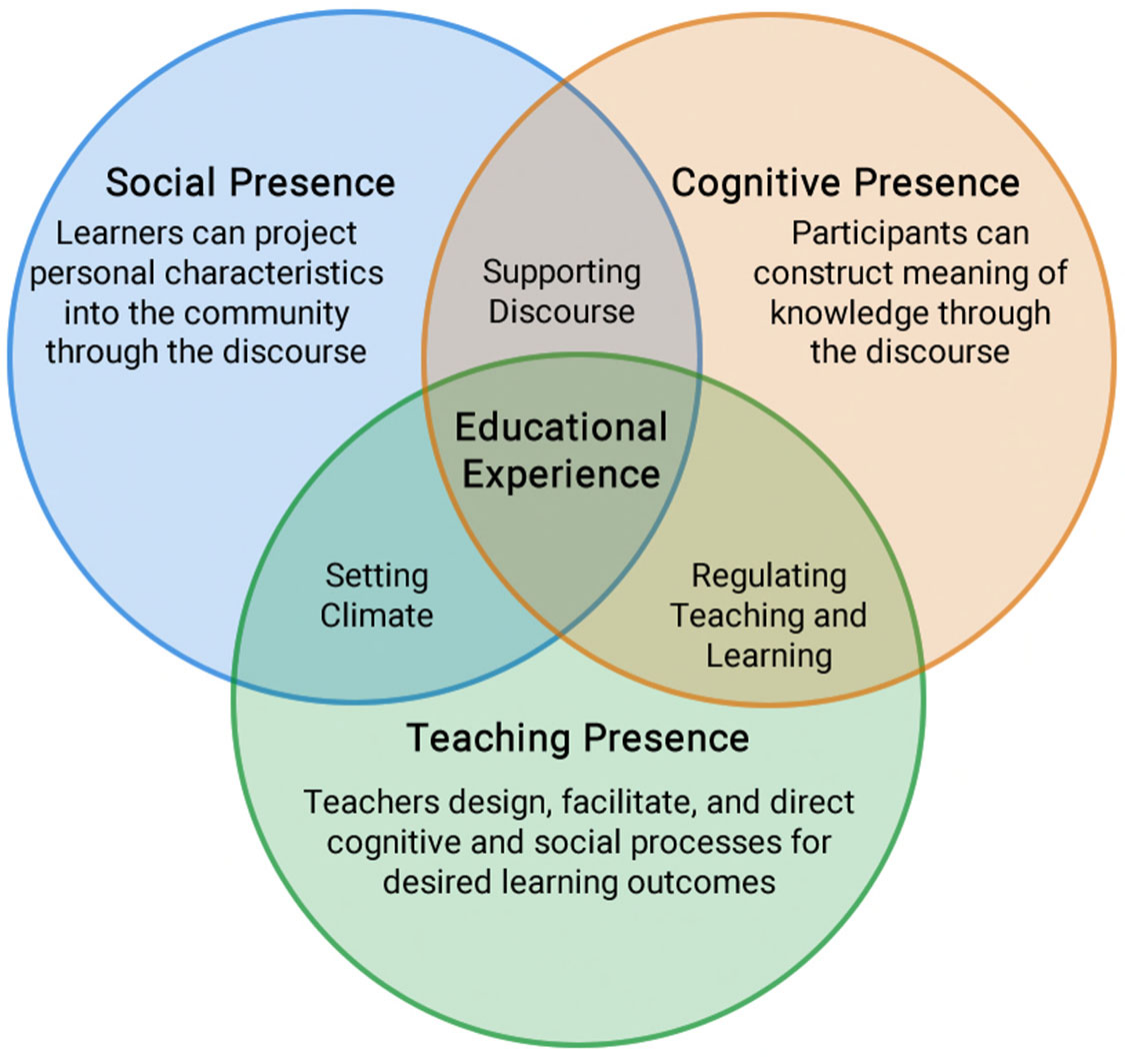
Community of Inquiry framework.

The Community of Inquiry ( Figure 1) posits that a sense of community is essential to effective learning environment and can be fostered through three highly inter-related core elements: cognitive, social, and teaching presences ( Garrison, Anderson and Archer, 1999). When learners feel safe and well supported to engage in collaborative learning discourse ( *social presence*), they actively participate in “shared creation and/or shared discovery” ( Schrage, 1995) and the co-construction of knowledge ( *cognitive presence*) ( Jorgensen, 2003). Educators play critical roles in creating both the social and cognitive presences by helping learners cross the threshold from feeling like an outsider to being an insider ( Wegerif, 2019), providing learners with opportunities to express their opinions in constructive ways, increasing the sense of community by allowing learners to moderate the inquiry, and developing curriculum accordingly ( *teaching presence*) ( Poole *et al.,* 2000).

(From ‘Critical Inquiry in a Text-Based Environment: Computer Conferencing in Higher Education’, by D. R. Garrison, T. Anderson and W. Archer, (2000), *The Internet and Higher Education, 2,* p. 88. Copyright 2000 by Elsevier Science Inc. Reprinted with permission.)

## Social Presence

*Social presence* marks a qualitative difference between a CoI and a simple process of downloading information from the internet. The virtual communication medium provides means for ‘social presence’ whereby learners can project themselves socially and emotionally as ‘real’ people (i.e., personal characteristics and identity) within the learning environment. The three main characteristics of social presence are *open communication, emotional expression,* and *group cohesion* ( Garrison, Anderson and Archer, 1999) *.*

## Tip 1: Create a virtual space where learners feel safe to engage in open communication

For an optimal virtual ASL experience, educators must offer a psychologically safe environment where ideas can be shared openly to promote collaborative learning.Psychological safety is defined as the perception of safety to engage in discourse without fear of embarrassment, rejection, or repercussion as the result of mutual trust and respect among team members ( Edmondson and Lei, 2014). Hence, collaboration should be questioning yet engaging, expressive yet responsive, skeptical yet respectful, and challenging yet supportive.

Examples of open communication include mutual awareness and respect for each other’s contributions. Mutual awareness is concerned with attending to the comments and contributions of others; it can be achieved by enabling learners to actively engage in messaging boards and online discussion forums. Respect for one another fuels the development and maintenance of relationships. Educators should encourage expressions of appreciation, agreement, and support of others using textual tools. Recognizing one another individually is critical, as non-verbal means of establishing and maintaining social interactions are not available in ASL ( Garrison, Anderson and Archer, 1999).

## Tip 2: Monitor for learners’ emotional expressions in the ongoing discourse

Encouraging expressed emotions can facilitate engagement. To optimize the discourse, educators need to monitor closely for indicators of emotional response and adapt the environments accordingly. Two examples of emotional expression that enhance discourse are humor and self-disclosure. Humor provides an invitation to start a conversation, aims at decreasing social distance, and conveys goodwill ( Gorham and Christophel, 1990), thereby contributing to the social presence and learning. Sharing one’s feelings, attitudes, experiences, and interests encourages others to be more forthcoming and to reciprocate. These observed learning behaviors reflect trust, support, and a sense of belonging within the CoI. Providing ASL users with opportunities to exchange personal information reduce their feelings of social isolation and allows them to form individualized perceptions of each other ( Shamp, 1991).

## Tip 3: Use social software to create authentic group cohesion without ‘being there’

Group cohesion is achieved when learners identify themselves as part of a group rather than merely as individuals. Cohesiveness induces a more involved critical inquiry and higher quality of discourse. Creating a forum for introductions (e.g., avatar profiles, greeting videos) and using ‘icebreaker’ assignments at the beginning of the course help learners feel welcome and encourage interactions. Off-topic forums using various social media platforms (e.g., Slack, WhatsApp group), video blog platforms (e.g., FlipGrid), or shared virtual workspace (e.g., Google suite) where learners can work collaboratively are readily available. Additionally, it is important to incorporate activities within the curriculum that build and sustain a sense of group commitment such as dividing and distributing work among students with preset objectives ( Anderson and Kanuka, 1997; Kanuka and Anderson, 1998).

Use of interactive and engaging technologies, termed *social software* ( Kesim and Agaoglu, 2007) *,* can enhance ASL *.*Examples are Flipgrid, Blackboard, and Microsoft Teams, which offer a virtual classroom wherein students can access course material, use discussion boards, and share resources. These platforms also allow for feedback from a moderator and peers and offer a means for effective communication that can foster social presence continuously, without shared time and space ( Johansen, 2017). Blogging or vlogging also allows individuals opportunities for thoughtful reflection and preparation prior to projecting themselves into the community. All of these platforms enable learners to be moderators who add content by way of personal reflection/experience to the discussion topic and, thereby encouraging them to move from being passive recipients of knowledge to active leaders.

## Tip 4: Design an environment that strategically puts learners in charge of their own journeys

Learning is optimized through construction of personal meaning. For instance, a story may resonate with learners more effectively than does theoretical content. Herczeg proposes methods for integrating experience design, interaction design, and instructional design to create challenging and motivating experiences in learning contexts ( Herczeg, 2004).

The ASL can include story telling (e.g., case study, short animated sequences) and provide activity spaces where learners themselves are part of the story (e.g., virtual case and role play). Learners must take an active role in developing and articulating their questions and goals of inquiry. Incorporating different views can be used as a simple strategy to personalize learning. Some learners prefer a sequential book-like view, but others may choose to access content via an application view (i.e., content categorized according to how it might be used). Creating different learning paths such as the use of a branching scenario or choose-your-own-adventure style can enhance learners’ engagement in a case-based learning ( Beck *et al.,* 2017; Mundy and Consoli, 2013). This type of learning can be achieved by using various online management platforms (e.g., “it’s learning”. “canvas”, “schoology” etc.) that not only allow creation of content suitable for different levels of learners, but also provide means for the educator to gauge learner’s comprehension, thereby enabling them to personalize the teaching for the individual learner at each step of learning process.

## Cognitive Presence

According to CoI, the element that is most basic to success in higher education is *cognitive presence.* It takes into consideration the extent to which the learners are able to construct and confirm meaning (of new knowledge) through sustained discourse within the CoI ( Garrison, Anderson and Archer, 1999). In team learning, learners demonstrate desirable learning behaviors (e.g., ask questions, seek feedback, experiment, and discuss errors or misconceptions), which expands and enhances the ASL ( Edmondson, 1999) **.**

## Tip 5: Implement concepts for acquisition of knowledge when creating instructional materials

Advances in learning sciences inform us that the acquisition of knowledge, skills, behaviors, and attitudes occur through establishing neural networks ( Doyle and Zakrajsek, 2013). This process involves three steps: *encoding,* the process of forming a mental representation of an external perception in short-term memory; *consolidation,* the transfer of important short-term memories to long-term memory; and *retrieval,* the movement, use, and change of long-term memories to short-term memory and back again ( Brown, Roediger and McDaniel, 2014; Doyle and Zakrajsek, 2013; Carey, 2014).

We recommend the following examples of instructional processes in ASL to optimize those three steps:

For *encoding,* we suggest implementing *distributed learning,* which refers to spacing out a specific content by dividing it into several study sessions offered over an extended period of time. While it is important to return to newly learned information within 24 hours of exposure ( Oakley, 2014), spacing sessions from days to weeks to months can reliably increase the retention for extended periods ( Doyle and Zakrajsek, 2013; Oakley, 2014). The ASL module should be designed to teach a body of information over the course of a few weeks or months to increase retention whenever logistically possible.

*Consolidation (also called “interleaving”)* involves mixing related but distinct material during study ( Carey, 2014). It forces the brain to reconcile previously learned information (principles, concepts, procedures) with more recent and upcoming information in the same learning activity ( Brown, Roediger and McDaniel, 2014; Oakley, 2014). The application of this concept requires deconstructing one subject (e.g., cardiac physiology) into small portions and then incorporating another set of small portions of related information or strategies (e.g., cardiovascular pharmacology). The process enables learners to seek or create connections among related parts and renders knowledge of content more cohesive and memorable. A distinct advantage of the interleaving strategy is that it forces a learner to understand “when” and “how” to use knowledge and skills in varying and unexpected circumstances ( Carpenter *et al.,* 2012; Oakley, 2014).

*Retrieval* involves using strategies to recall knowledge that was learned previously. Retrieval practices force movement of information between short- and long-term memory, leading to improved comprehension and retention ( Van Hoof and Doyle, 2018). The ASL modules can offer periodic quizzes within a module or between the modules as one of the means for offering practice in retrieval.

## Tip 6: Design a multimedia learning experience using the “Less is More” approach

The design and development of instructional materials for ASL and their regulation or flow of new information into the learner’s working memory are often associated with cognitive load theory ( Paas *et al.,* 2003), which explains a learner’s limited cognitive processing capacity needed to acquire new knowledge and skills. Mayer’s principles of multimedia learning serve as a useful guide for educators to address cognitive loads within instructional design ( Mayer, 2009). Managing *germane cognitive load* (i.e., the results of cognitive process that contributes to, rather than interferes with, learning), reducing *extraneous cognitive load* (i.e., the results of instructional processes that require leaners to engage in working memory activities not directly related to learning), and employing *social cues* can improve learning outcomes from lecture videos ( Mayer, 2009; Mayer and Pilegard, 2014; Mayer and Fiorella, 2014; Paas and Sweller, 2014).

Examples of better practices that can be used to achieve these goals are:

*Segmenting -* Creating learner-paced segments is called *segmenting.* Mayer found that when learners can control the pace of their learning, they perform better on recall tests ( Mayer, 2009). Pacing can be provided by adding ‘next’ buttons or allowing the learner to determine the speed at which a video plays.

*Pre-training principle -* Knowing the names, terms, and characteristics associated with a concept before being introduced to its content allows learners to gain a better understanding from a multimedia lesson. It can be achieved by using familiar names and terms and/or by creating an introductory guide.

*Coherence principle -* Learning is best achieved when extraneous, distracting materials are minimal. We recommend using simple texts and visuals that are related directly to the learning topic.

*Signaling principle -* Highlighting essential information can help people learn better. Using features such as highlighting important words and using animated arrows to point out significant information helps learners acquire knowledge.

## Tip 7: Use multimedia that fit the purpose

A variety of interactive modalities are available to create and deliver content for ASL. Using the practical inquiry model ( Dewey, 1933), various multimedia can be used to repurpose content for improving cognitive presence and enhance communication, engagement, and sense of community among educators and learners.

Examples of media that serve as triggering events for a CoI include SurveyMonkey and Google forms, wherein concepts can be introduced in a question-based format that allows learners to gauge their basic understanding of the concepts. These media can also be used for a case-based learning format such as a choose-your-own-adventure ( Beck *et al.,* 2017), which allows learners to apply the information gained in an interactive format. ‘Powtoon’ and ‘Toonly’ can serve as efficient media for exploring content by presenting information in an animated format that enhances the learner’s engagement. For the purpose of deliberate practice, often difficult to teach in ASL, a medium such as VoiceThread can allow learners to view, learn, and practice certain skills at their own pace. After practicing, learners can upload a video of the exercise to the platform and receive feedback from a course instructor via an editing tool provided on this platform.

Using video to deliver curricular content is an integral part of ASL that requires thoughtful consideration. The two predominant components of video style in ASL are *human embodimen*t (virtual representation in the form of a talking head, a virtual hand, etc.) and *digital media* (depicting data using slides, animations, simulation, etc.). Beyond these, common characteristics of highly effective instructional videos include fast-paced, high-definition production, static images and animation, and highlighted text. Among the various style options for online videos that are described in the literature, learning glass and demo video were highly rated for student satisfaction and engagement, despite no statistical differences in learning outcomes across different video styles ( Choe *et al.,* 2019).

Additional online learning activities such as word puzzles, jeopardy, audio flashcards, or quizzes can be created by on Quizlet, Jeopardy Labs, QStream StudyStack to keep learners engaged.

## Teaching Presence

*Teaching presence* is defined “as the design, facilitation, and direction of cognitive and social processes for the purpose of realizing personally meaningful and educationally worthwhile learning outcomes” ( Anderson *et al.,* 2019). It is thought to be the unifying element that brings the learning community together, enabling the cognitive and social aspect of online learning ( Garrison and Archer, 2000; Garrison and Cleveland-Innes, 2005). Through adequate teaching presence, formal learning that facilitates personally relevant and educationally defined outcomes can be achieved (Anderson *et al.,* 2001). An ASL is not merely communication between an educator and learners using online media; rather, it involves course readings, exploration of content, exercises, and individual and collaborative projects. Thus, teaching presence includes facilitating the discourse and providing direct instructions to learners to help them clarify misconceptions and solidify learning. It is imperative to harness one’s teaching perspective while maintaining teaching presence in ASL, given the lack of direct interaction with the learners. Pratt’s teaching perspectives can serve as an efficient foundation for facilitating discourse in ASL ( Pratt and Collins, 2020) ( Table 1).

**Table 1:** Pratt’s teaching perspectives with description and potential example of its use in ASL.

Pratt’s Teaching Perspective	Description	Examples in ASL
Transmission	Instructors have mastery over their content The content is presented accurately and efficiently It is learner’s responsibility to master the content	Create didactic videos using animations Use Bloom’s taxonomy to create modules
Apprenticeship	Teaching and learning occurs by observing in action Learners must be engaged in authentic tasks and relationships	Create videos while performing tasks Ask students to upload videos of themselves performing the same tasks and providing feedback Use deliberate practice
Developmental	Learner’s reasoning or thinking are developed by fostering the growth of complex and sophisticated cognitive structures	Use multimedia platforms involving case-based learning Use gamification theory
Nurturing	Developing a trusting learner-teacher relationship is essential Respect the learner’s self-concept and self-efficacy	Use platforms such as VoiceThread to guide and provide feedback on certain tasks
Social Reform	Pursuit of social change is more important than individual learning	Develop modules on impact of race and socio-economic status in health outcomes

## Tip 8: (Re)Design and/or (Re)organize the curricular content

In ASL, much of the learned expectation of classroom norms is not available for use by either the student or the educator, requiring that educators be more explicit and transparent in their planning process. When designing curricula, educators must pay particular attention to descriptive details of the process, structure, evaluation, and interaction of components of the curriculum ( Laurillard *et al.,* 2000). For instance, curricular content may need to be reconfigured andreorganized to provide more interactive sessions that stimulate increased engagement of the learners *.* Didactic materials may need to be converted into various types of exercises that engage the learner and require responses. Depending on the content, educators may need to convert oral discourse to videos, animations, games such as matching icons, and drawings. Presenting content in a conversational rather than an academic style will help increase the learners’ engagement.

When building or designing the curricular content, it is imperative to provide clear instructions for completing the course, along with guidance for use of technology. The following questions should be addressed ( Fink, 2003):

1. What are the learning goals for the set discourse?
2. What are the feedback and assessment methods to ensure that learning goals are being achieved?
3. What teaching/learning activities need to be done by educator and learner to achieve set learning goals?

Finally, it is imperative to communicate expectations for educators’ participation (e.g., involvement in asynchronous discussion and email response times), as well as expectations of students’ participation and activities, including timelines for group activities. The educator also provides information on the project work upfront and organizational service to students by providing guidelines and tips and modeling appropriate etiquette and effective use of the medium.

## Tip 9: Facilitate the discourse among leaners

Facilitating discourse is critical to maintaining the interest, motivation, and engagement of learners. It is certainly a challenging aspect in ASL. The role of a facilitator is not only to support and encourage participation by modeling appropriate behaviors, but also to focus on creating meaning and confirming understanding. The educator fosters fruitful discussion, often sharing personal experiences, while challenging and testing students’ ideas (e.g., asking for clarification/elaboration). ASL inherently runs the risk of having the discussion dominated by certain participants, so the educator must remain mindful of commenting upon and encouraging student responses equally, while drawing in the less active participants. Finding consensus or agreement and summarizing class discussions help students to reinforce goals and objectives ( Lowenthal and Parscal, 2008).

Pratt’s teaching perspectives can help educators identify various individual styles for facilitating discourse. For example, educators identifying with Pratt’s *apprenticeship* perspective, wherein they engage learners in authentic tasks, can provide videos of themselves performing certain tasks (e.g., auscultation and history-taking), thereby allowing students to model behavior and providing opportunities for informed discussions. Educators who have a *developmental* perspective as the dominant trait according to Pratt’s teaching inventory, wherein they develop learners’ reasoning or thinking by fostering growth of complex and sophisticated cognitive structures, can use multimedia platforms involving case-based learning with reasoning to engage students in lively discourse. ASL also can serve as a valuable option for the *social reform* perspective of Pratt’s teaching inventory. By engaging learners and challenging the status quo of certain aspects of medical education (e.g., impact of race and socio-economic status in health outcomes), educators can initiate meaningful expansion of students’ perspectives in a safe environment.

## Tip 10: Oversee and direct the instruction

A primary responsibility of educators is to provide intellectual and scholarly leadership and share their knowledge of the subject matter with their students. The instructor must be able to set and communicate the intellectual climate of the course and model the qualities of a scholar ( Davie, 1989). The role of the educator, in any context, involves having pedagogical expertise and explaining the subject matter. Educators also keep the discussion focused by directing attention to particular concepts and information that are necessary to frame or pursue knowledge. Direct instruction takes the form of statements that confirm understanding through assessment and explanatory feedback. Diagnosing misconceptions is another critical task of the on-line educator. Often students hold misconceptions that impair their capacities to build more correct conceptions and mental schemata. Although the design of effective learning activities should open opportunities for students themselves to uncover these misconceptions, the educator’s expertise, comments, and questions as direct instruction are critical to the dissemination of accurate information.

## Cautionary Tips: Using Technology and Not Letting Technology Use You

Integration of technology often is marred by the continuous evolution of resources and varying needs across curricular content and contexts. Not uncommonly, using technology becomes a goal rather than a means to an educational result. When considering how to use technology for ASL, it is important to evaluate the role of technology - is it content delivery, content creation, interaction, or other? The answers to these questions will enable educators to choose the appropriate platforms, as the needs will vary depending upon the educational context.

## Tip 11: Set clear goals for what you want to achieve in the virtual transition

Various technology models can help transform content for ASL. Examples are Substitution - Augmentation - Modification - Redefinition (SAMR); Technology Integration Planning (TIP); Technology Integration Matrix (TIM), Technology, Pedagogoy and Content Knowledge (TPACK); and Replacement - Amplification - Transformation (RAT) ( Kimmons, Graham, and West, 2020).

While no one model sufficiently satisfies various educational contextual needs, the PICRAT model suggested by Kimmons *et al.* ( Kimmons, Graham, and West, 2020) is a student-focused, pedagogy-driven model that can be effective for systematic planning of an ASL. It is based on a three-level response metric and answers two important questions: “What are students doing with technology?” and “How does this use of technology impact the educator’s pedagogy?” PIC (passive, interactive, and creative) answers the first question and RAT (replacement, amplification and transformation) answers the second question.

### PIC: Passive, Interactive, Creative (Students’ Perspectives)

Educators often use technology for passive delivery of knowledge (e.g., PowerPoint presentations or videos), leading to passive involvement of students. Incorporating interactive learning by means of exploration, experimentation, and collaboration (e.g., use of computerized adaptive tests, simulations, digital flash cards, etc.) allows students to interact directly with technology. It is important to remember that potential for interaction does not mean interactive learning. For creative use of technology, students can use video editing, sound mixing, and presentation creation thus allowing students to create new content based on their understanding.

### RAT: Replacement, Amplification, Transformation (Teacher’s Pedagogy Perspective)

Often educators use technology to *replace* a previous teaching format, with no functional improvement in their practice (e.g., digital flashcard for paper flashcard, electronic slides for overhead projector, interactive whiteboard for a chalkboard). While this change can be useful in certain educational contexts, there often is no justifiable advantage to students’ learning from use of technology.

In comparison, *amplification* is use of technology to improve learning outcomes or practices (e.g., using review feature of google docs to provide more efficient and focused feedback, using VoiceThread platform to provide digital feedback [i.e., audio, video] on a presentation or clinical skills video performed by student) for a mobile device.

*Transformation* uses technology to enhance pedagogical practices enacted. Use of technology can transform the instructional method, the learning processes, and/or the actual subject matter. Innovative applications of technology to engage learners in problem-solving allow for restructuring and reorganizing learning in previously unimaginable ways ( Pea, 1985; Puentedura, 2010). For example, video blogs support and build collaborative communities, and interactive videos personalize learning through options and choice.

### The PICRAT Matrix

**Figure 2.**
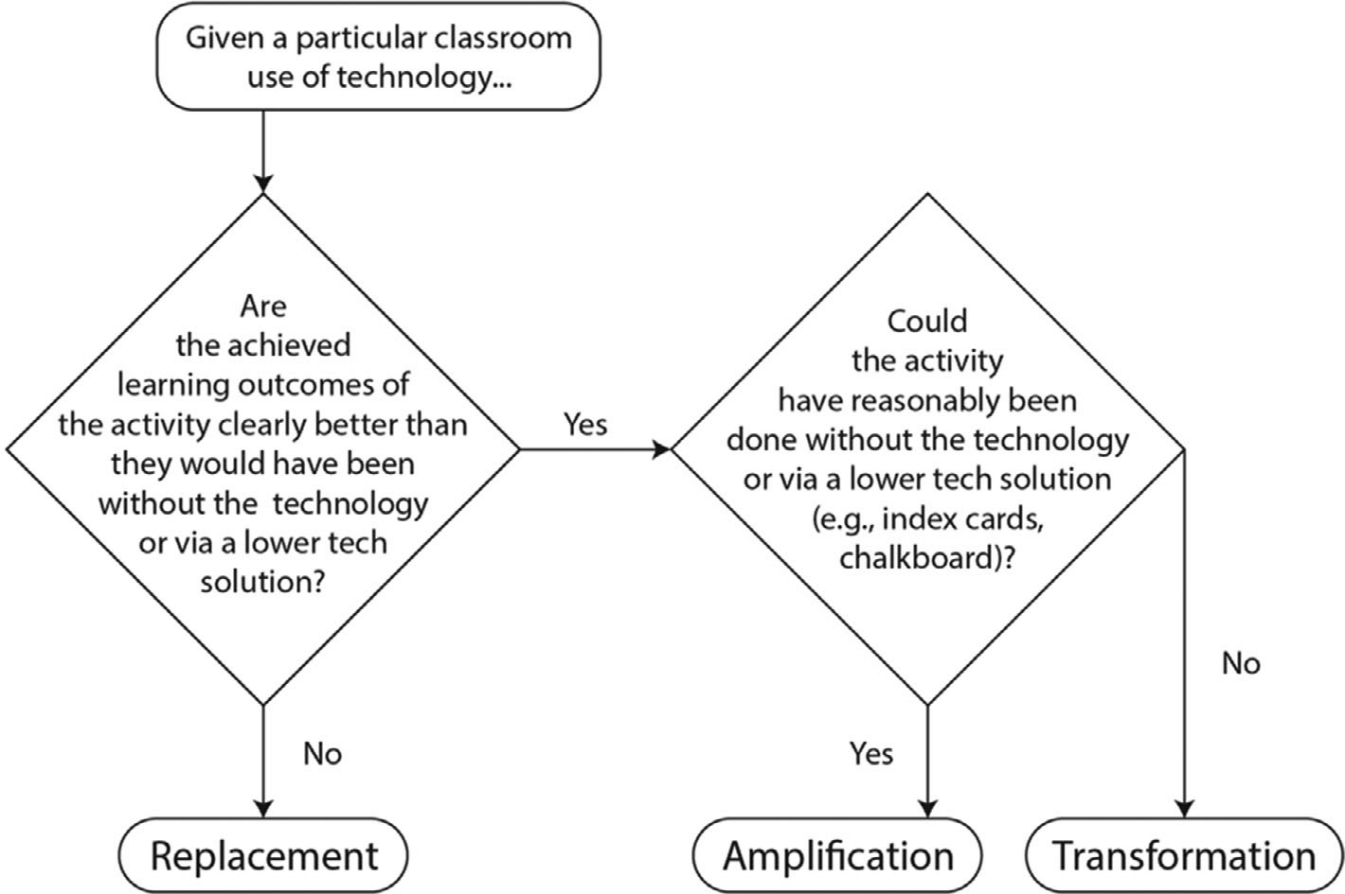
Series of questions to discriminate Replacement, Amplification and Transformation.

**Figure 3.**
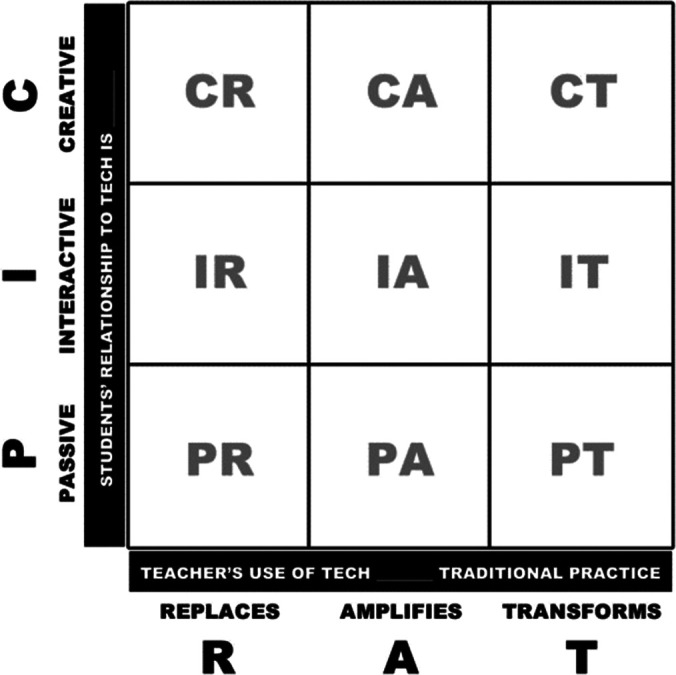
The PICRAT Matrix-Passive/Interactive/Creative and Replace/Amplify/Transform.

**Figure 4.**
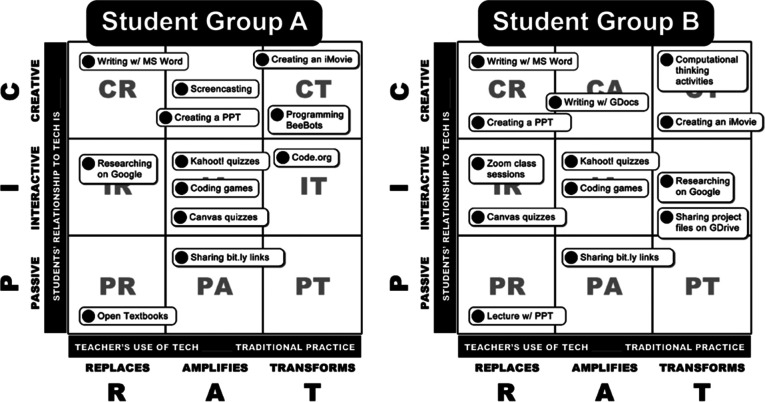
Two examples of potential use of the PICRAT matrix in different scenarios.

When considering the use of technology, Kimmons *et al.* ( Kimmons, Graham and West, 2020) suggest asking a series of questions **( Figure 2)** to determine if the use of technology is for replacing or improving learning. If it is for enhanced learning, then further differentiation between amplification and transformation is important. The PICRAT matrix **( Figures 3 and 4)** offers a structured approach for a purposeful and transparent planning of an ASL. Educators could use this matrix to decide which of the nine possibilities one aims to achieve, plan a balanced engagement between learners and technology, or determine whether one has capitalized on technology integration.

(From Kimmons, R., Graham, C. R., & West, R. E. (2020). ‘The PICRAT model for technology integration in teacher preparation. Contemporary Issues in Technology and Teacher Education’, 20 (1), 176-198. Reprinted with permission.)

(From Kimmons, R., Graham, C. R., & West, R. E. (2020). ‘The PICRAT model for technology integration in teacher preparation. Contemporary Issues in Technology and Teacher Education’, 20(1),176-198. Reprinted with permission.)

(From Kimmons, R., Graham, C. R., & West, R. E. (2020). ‘The PICRAT model for technology integration in teacher preparation. Contemporary Issues in Technology and Teacher Education’, 20(1), 176, 20(1), 176-198. Reprinted with permission.)

## Tip 12: Stay true to your teaching identity and dominant perspectives

Educators develop their own pedagogical identities, which often are based on perspectives or images of what constitutes effective teaching. When educators are required to transition to a different format of teaching, including ASL, a tendency is to question or doubt their pedagogical identities. For example, an educator who teaches by developing a learner’s reasoning or thinking by challenging them with complex and sophisticated structures might find it difficult to gauge the learner’s level of understanding due to lack of face-to-face interaction. This questioning can lead to identity dissonance as an educator, resulting in frustration and low self-worth ( Monrouxe, 2010). To avoid this incongruence with one’s teaching identity, it is important for an educator to undertake critical self-reflection to recognize and mitigate identity dissonance.

Self-reflection can be guided by undertaking Pratt’s teaching perspectives ( Pratt and Collins, 2020), which can enable educators to identify the components of teaching that define them so they can remain central to their teaching, regardless of the educational platform used. By exploring and understanding teaching perspectives, educators can critically assess their personal values and beliefs about teaching, allowing them to explore new roles and strategies, and can enable them to build competence and self-efficacy in a new educational context. This self-reflection exercise also can help identify one’s own strengths and weaknesses, thereby helping to determine how the use of new online/ASL platforms can enhance the learning experience for themselves and the students without challenging one’s teaching identity ( Maggio *et al.,* 2018).

## Take Home Messages

• Use of Community of Inquiry framework can help with planning and development of asynchronous curricula
• Use of PICRAT model can serve as the basis to use technology to reform the curriculum and learning
• Use of technology should be a means to an end, not the end itself
• It is important for the educator to have an interactive role to oversee instruction, facilitate discourse, and ensure all students are engaged
• While ASL can be challenging for both educator and learner, it is important to stay true to one’s teaching identity.

## Notes On Contributors

**Surbhi Maheshwari** - is a former Assistant Professor, Department of Pediatrics, Section of Neonatology, Baylor College of Medicine and Texas Children’s Hospital.

**Parag Jain** - Assistant Professor, Department of Pediatrics, Section of Critical Care Medicine, Baylor College of Medicine and Texas Children’s Hospital.

**B. Lee Ligon** - Instructor/Department Editor, Department of Pediatrics at Baylor College of Medicine. Managing Editor *Pedi Press* and Editor/graphics of the Center for Research, Innovation and Scholarship in Medical Education. She is a retired English professor at a private university, where she created and launched the professional writing program.

**Satid Thammasitboon** - Associate Professor, Director of Center for Research, Innovation and Scholarship in Medical Education, Department of Pediatrics, Section of Critical Care Medicine, Baylor College of Medicine.
