# Supplementary material for: Twelve Tips for Creating and Supporting a Meaningful Asynchronous Learning as Parts of Virtual Transition of a Curriculum
Source: MedEdPublish (2016). 2021 May 5;10:111. [Version 1] doi: 10.15694/mep.2021.000111.1 (PMC10939517; doi:10.15694/mep.2021.000111.1)
Supplement: Peer review history

# Reviewer report: Reviewer response for version 1

**DOI:** 10.21956/mep.18967.r27158

**Published:** 2021-07-09

**Authors:** John Sandars

**Affiliations:** 1 Edge Hill University

This review has been migrated. The reviewer awarded 4 stars out of 5 These 12 Tips will provide an excellent practical guide for all medical educators that is based on the author's practical experience and supported by some key references. It is a large topic but overall, a great summary.

Reviewer Expertise:

NA

No decision status is available

# Reviewer report: Reviewer response for version 1

**DOI:** 10.21956/mep.18967.r27157

**Published:** 2021-06-18

**Authors:** Barbara Jennings

**Affiliations:** 1 Norwich Medical School

This review has been migrated. The reviewer awarded 4 stars out of 5 This is a very well written article, with the benefit of a thorough bibliography. The tips and cited literature will be of interest to all clinical educators who deliver and design e-learning and particularly lessons, modules, and courses with asynchronous learning activities.I will recommend it widely within my department and beyond. The article is timely. The use of asynchronous teaching and learning accelerated greatly during the pandemic (and before, in response to expanding cohorts of clinical trainees with an associated need for flexible timetables of clinical placement opportunities) and now educators will be ready to reflect on and develop their expertise in this aspect of teaching. The tips listed go far beyond the notion of replacing face to face (F2F) experiences with something similar and discussions of “amplification”, enhanced learning, and “transformation” with reference to the PICRAT model are welcome.One strength of this article is its organisation, with the subdivision of the tips according to categories in the conceptual framework e.g. social and cognitive presence. Also, the descriptive sub-headings used for each tip are helpful. The underpinning scholarship in several sections gives the reader access to core concepts about knowledge acquisition and retention (e.g. see tips 5 and 6) – which is helpful for clinical faculty development.I found myself saying “hear, hear” when I read tips 2 to 4 – I have used whiteboard animations to bring humour, signposting and storytelling in my own MOOC and e-learning modules – these also facilitate “human embodiment” if the virtual hand resembles that of the teacher. I use these sorts of recordings in welcome videos and for case-based genetics vignettes to trigger knowledge activation/retrieval and to promote engagement with new material. I would be interested to know what sorts of ASL courses the authors have developed and delivered – what experiences informed these guidelines?My main gripe with the article is the promotion of learning style inventories (Pratt’s teaching perspectives) without any evidence presented for the validity/utility of their application in course design. I agree it is satisfying and helpful for a teacher to be a visible and share their identity - but I think most aspects of teaching presence listed in table 1 will be expressed in a good asynchronous class/module. The precise aspect depends on the context – is a predominant style-category important information or perhaps a limiting construct? Perhaps this could be justified if a revised version of the article is produced.A minor point is the lack of citations for some software described.

Reviewer Expertise:

NA

No decision status is available
